# Triadin Decrease Impairs the Expression of E-C Coupling Related Proteins in Muscles of MPTP-Induced Parkinson’s Disease Mice

**DOI:** 10.3389/fnins.2021.649688

**Published:** 2021-04-22

**Authors:** Min Hyung Seo, Sujung Yeo

**Affiliations:** ^1^Department of Korean Medicine, Sangji University, Wonju, South Korea; ^2^Research Institute of Korean Medicine, Sangji University, Wonju, South Korea

**Keywords:** TRDN, Parkinson’s disease, Ca^2+^ channel, MPTP, MPP+, skeletal muscle, C2C12

## Abstract

Parkinson’s disease (PD), caused by destruction of dopaminergic neurons in the brain, leads to motor symptoms like bradykinesia, tremor, and walking impairments. While most research effort focuses on changes in neuronal pathology we examined how muscle proteins were altered in a 1-methyl-4-phenyl-1,2,3,6-tetrahydropyridine (MPTP) mouse model of PD. A Ca^2+^ release channel complex, consisting of ryanodine receptors (RYR), triadin (TRDN), and calsequestrin (CSQ1), is important for excitation-contraction coupling in the sarcoplasmic reticulum membrane in muscles. Thus, we investigated changes in the RYR Ca^2+^ release channel components in PD mice model. Based on a report that TRDN deletion impairs skeletal muscle function, we also investigated how the knock-down of TRDN affects other components of the RYR channel in the PD model. In this study, the expression levels of the components of RYR channels decreased in the quadriceps femoris muscle of MPTP-induced PD mice and in C2C12 cells treated with 1-methyl-4-phenylpyridinium. We show that decreased TRDN levels decrease RYR and CSQ1 levels. These results suggest that the levels of proteins related to Ca^2+^ channel function decreased in this model, which could impair muscle function. We conclude that muscle function alterations could add to the bradykinesia and tremor in this model of PD.

## Introduction

Parkinson’s disease (PD) is a brain disorder that is caused by the degeneration of dopaminergic cells in the substantia nigra (SN). The symptoms of PD include bradykinesia (slow movement), tremor, muscle rigidity, postural instability, and walking impairments. The symptoms related to the neuromuscular function are mainly observed in patients with PD. PD usually occurs in elderly people and causes dangerous situations like falling which can cause secondary injury. Alleviating the symptoms related to muscles will improve the patients’ quality of life. Furthermore, it was shown that bradykinesia could be due to insufficient recruitment of muscle force for the initiation of movement (Berardelli et al., 2001). Therefore, in this study, we focused on muscle function in PD and investigated factors related to Ca^2+^ channels in muscles.

Motor neurons in contact with skeletal muscles signal the muscle to contract by releasing acetylcholine into the synaptic cleft. Acetylcholine depolarizes the muscle fiber and triggers an action potential in the muscle fiber membrane, which travels to the transverse tubule (T-tubule). This action potential moves to the sarcoplasmic reticulum (SR), which has Ca^2+^ channels that release Ca^2+^ into the cytoplasm to enable muscle contraction. When the level of Ca^2+^ in the cytoplasm is high, the muscle contracts by binding Ca^2+^ to the troponin complex (Campbell et al., 2010; Hudspeth et al., 2013).

Muscle contraction is regulated by Ca^2+^ levels in the cytoplasm, which in turn are related to Ca^2+^ channels. Ryanodine receptors (RYRs), calsequestrin (CSQ1), triadin (TRDN), junctin, and dihydropyridine receptors are proteins involved in Ca^2+^ homeostasis (Oddoux et al., 2009) and excitation contraction (E-C) coupling (Sutko and Airey, 1996). In this study, we focused on the role of TRDN in muscles. Furthermore, proteins such as RYRs and CSQ1s, which are related to TRDN in the SR membrane Ca^2+^ channel, were studied.

Triadin has been mostly studied in the cardiac and skeletal muscles. TRDN regulates Ca^2+^ release from the SR. In the cardiac muscle, *TRDN* mutations trigger the leakage of Ca^2+^ ions from the SR lumen because CSQ1 is unable to inhibit the release of Ca^2+^ ions by RYR (Roux-Buisson et al., 2012). TRDN deletion impairs muscle function (Oddoux et al., 2009). In addition, although CSQ1 has a role as a luminal calcium sensor for RYR (Beard et al., 2005), TRDN is also able to sense the Ca^2+^ level in the SR lumen by mediating interactions between RYR and CSQ1 (Zhang et al., 1997).

In this study, TRDN-related factors were studied in a 1-methyl-4-phenyl-1,2,3,6-tetrahydropyridine (MPTP)-induced PD mouse model (Brady et al., 2005). The quadriceps femoris (QF) muscle was used because the QF is the most important muscle for walking and jumping. The mouse model was a semi-chronic model induced by 4 weeks of MPTP administration. The C2C12 cell line, which is an immortalized mouse myoblast cell line, was used to determine the change in TRDN-related factors after treatment with 1-methyl-4-phenylpyridinium (MPP+) and TRDN siRNA.

We hypothesized that Ca^2+^ channels related to muscle contraction may be impaired in PD. The purpose of this study was to determine the association between the Ca^2+^ channel components, especially TRDN, and PD. Furthermore, this study could contribute to advancing the research on the mechanism of bradykinesia, one of the symptoms of patients with PD.

## Materials and Methods

### MPTP-Induced PD Mouse Model

Six-week-old male C57BL/6 mice (20–22 *g*; DBL, Korea) were divided into two groups: the control group and MPTP-treated group (MPTP). In the control group, mice were injected intraperitoneally with 100 μL phosphate-buffered saline (PBS) once a day for 4 weeks, while in the MPTP group, mice were injected intraperitoneally with MPTP-HCL (20 mg/kg of free base; Sigma, United States) in PBS (100 μL) every 24 h for 4 weeks to produce a semi-chronic model of PD. On the day after the final MPTP treatment, mice were anesthetized using Alfaxan and perfused transcardially with cold PBS for western blotting. The MPTP model of PD shows dopaminergic cell death in the SN (Choi and Lim, 2010; Yeo et al., 2013). All animal experiments conducted for this study were approved by the Sang Ji University Animal Experimentation Committee.

### Rotarod Test

Rotarod tests were performed to evaluate the motor ability of MPTP mice before the last MPTP injection. Training was performed in the second week for 2 days at 30 rpm for 15 min, once a day. The rotarod treadmill diameter was 280 mm, and the test was performed in an accelerated mode for 4 min from 10 rpm to 50 rpm in 5-min running time. After 4 min of accelerated mode, 50 rpm was maintained for 1 min until completion. The time until the first fall or the first drop was measured. The test was repeated two times and the measurement of last test was used as results for statistical analysis (*n* = 5/group).

### Pole Test

The pole test was conducted to evaluate the motor ability before the last MPTP injection. In the pole test, a wooden vertical pole (length 548 mm, diameter 8 mm) was used. The time the mouse took while moving from the top to the bottom of the pole was measured. Training was performed in the second week for 2 days, once a day. The test was performed two times and the measurement of last test was used as results for statistical analysis (*n* = 5/group).

### Immunohistochemistry

The brains of MPTP-induced PD mice were perfused with 4% paraformaldehyde and fixed in 4% paraformaldehyde for 1 day at 4°C. After fixation, the brains were dehydrated with 30% sucrose buffer for 2 days at 4°C. Coronal sections (40 μm) were cut using a cryomicrotome. Immunohistochemical analysis was performed using an ABC kit, a Mouse on Mouse (M.O.M) immunodetection kit (Vector Laboratories, CA, United States), and a modification of the avidin-biotin-peroxidase method. Sections encompassing the striatal and SN regions were incubated in 3% H_2_O_2_ with PBS (pH 7.4), and then incubated in blocking buffer [1% bovine serum albumin (BSA), 10% horse serum in PBS]. When the mouse anti-tyrosine hydroxylase (TH) antibody (1:200; Santa Cruz Biotechnology, United States) was used, tissues were treated with an M.O.M mouse Ig-blocking reagent (Vector Laboratories, CA, United States) at room temperature for 1 h before incubation with the primary antibody overnight at 4°C. Thereafter, the sections were treated with a biotinylated anti-mouse IgG and an avidin-biotin-peroxidase complex, which reacted with diaminobenzidine-hydrogen peroxide. Dopaminergic neuronal cells were analyzed using a Nikon X-cite series 120Q microscope (Nikon, Japan).

### Western Blotting

The QF muscle tissue was homogenized in 20 mM radioimmunoprecipitation assay buffer using a sonicator (Qsonica Q55, United States) on ice for 20 min.

C2C12 cells were incubated and homogenized in Tris–Triton cell lysis buffer (GenDEPOT, United States) for 20 min on ice.

After the tissues and the C2C12 cells were centrifuged at 12,000 rpm at 4°C for 15 min, supernatant samples were separated using 4–15% sodium dodecyl sulfate-polyacrylamide gel electrophoresis and then transferred to polyvinylidene difluoride membranes (Pall Life Science, United States). The membranes were blocked with 3% BSA at room temperature and then incubated with primary antibody overnight and washed with 0.1% Tris–buffered saline with Tween 20. The membrane was also incubated with a secondary antibody for 1 h and washed with 0.1% Tris–buffered saline with Tween 20.

Rabbit anti-TRDN (1:2000, Cloud-Clone Corp., United States), mouse anti-CSQ1 (1:500, Santa Cruz Biotechnology, United States), mouse anti-RYR (1:500, Santa Cruz Biotechnology, United States), and mouse anti-β actin (1:5000; Santa Cruz Biotechnology) antibodies were used as primary antibodies.

### Immunofluorescence

Longitudinal QF muscle cryosections were used for immunofluorescence in the control and MPTP groups. The sections were fixed in 4% paraformaldehyde and methanol. After fixation, the sections were incubated in blocking buffer (1% BSA, 5% goat serum in PBS) for 1 h. The samples of both groups were incubated with the primary antibodies, mouse anti-RYR (1:200, Invitrogen, United States) or mouse anti-CSQ1 (1:200, Invitrogen, United States) and rabbit anti-TRDN (1:200, Cloud-Clone Corp., United States), followed by secondary antibodies, goat anti-mouse IgG (H + L) fluorescein isothiocyanate (FITC)-conjugated (CUSABIO, United States), and goat anti-rabbit IgG (H + L) tetramethylrhodamine (TRITC)-conjugated (Novex, United States). Finally, DAPI (1 μg/mL) was used to label the cell nuclei. Photographic documentation was performed using a Nikon X-cite series 120Q microscope (Nikon, Japan). The exposure parameters were the same for the control and MPTP groups.

C2C12 cells were fixed in 4% paraformaldehyde and blocked for an hour. Rabbit anti-TRDN (1:200, Cloud-Clone Corp., United States) was used as the primary antibody, followed by a secondary antibody, goat anti-rabbit IgG (H + L) TRITC-conjugated (Novex, United States). Thereafter, DAPI (1 μg/mL) was used to label the cell nuclei. The control and MPP + group staining procedures were performed concurrently.

### Cell Lines and Cultures

C2C12 cells from the mouse-engineered myoblast cell line were cultured under standard culture conditions (5% CO_2_, 37°C). Dulbecco’s modified Eagle’s medium (BioWest, United States) containing 10% fetal bovine serum (GenDEPOT, United States) and 100 U/mL of penicillin-streptomycin (Gibco, United States).

### MPP + Treatment

C2C12 cells were treated with 1, 2.5, or 5 mM MPP + iodide (Sigma) for 18 h. MPP + was administered at the same time in each experiment.

### Short Interfering RNA Knockdown

C2C12 cells were incubated in Opti-MEM medium (Gibco, United States) at least 1 day before siRNA (*short interfering RNA*) transfection. The transfection reagent and TRDN siRNA were applied (3.5:1) when the density of C2C12 cells was 30%. Transfection was continued for 48 h. siRNA against TRDN (5-UC AUG UGG GUA GAC UCA GU-3) and negative control duplexes (5-UUC UCC GAA CGU GUC ACG UTT-3) were used (Bioneer Inc., South Korea).

### Imaging Software

ImageJ software developed at the National Institutes of Health and the Laboratory of Optical and Computational Instrumentation (University of Wisconsin) was used to analyze the images.

### Statistical Analysis

Statistical analyses were carried out with Student’s *t*-test and analysis of variance in SPSS 25 (SPSS Inc. Released 2017, PASW Statistics for Windows, Version 25.0, United States). All values are expressed as mean ± standard error.

## Results

**FIGURE 1:**
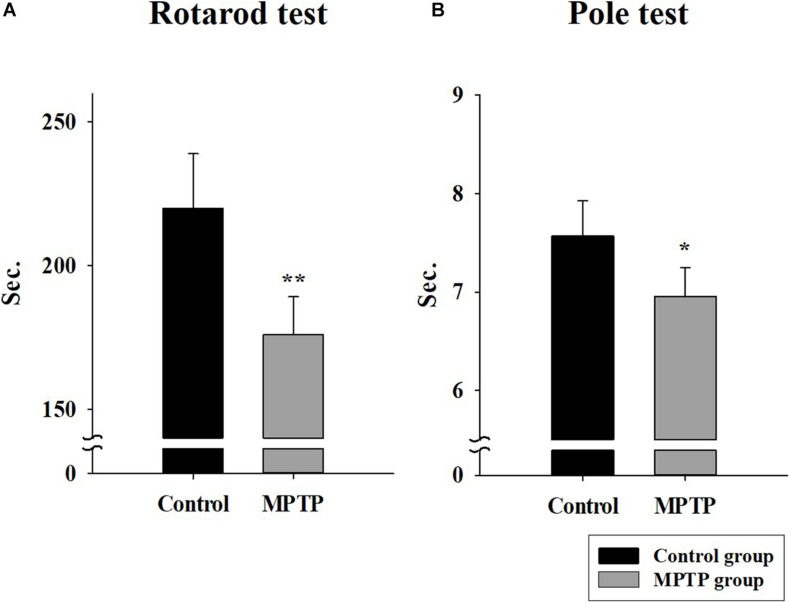
Rotarod and pole tests in the control group and the 1-methyl-4-phenyl-1,2,3,6-tetrahydropyridine (MPTP)-induced Parkinson’s disease group. **(A)** The rotarod (28 mm diameter) test was conducted in Accel Forward mode for 4 min from 10 to 50 rpm during 5 min of running time. The final minute was carried out at constant 50 rpm. (*n* = 5, **P < 0.005) **(B)** Pole test. The length and diameter of the pole were 548 mm and 8 mm, respectively. The mouse ran from the top to the bottom of the vertical wooden pole. (*n* = 5, *P < 0.05) Statistical analyses were carried out with Student’s *t*-test.

The MPTP-induced PD mouse model was created by injecting MPTP-HCL (20 mg/kg) every 24 h for 4 weeks. To check the status and motor ability of mice in the control and MPTP groups, rotarod and pole tests were performed. The results are shown in Figure 1. In the rotarod test, the mice in the MPTP group fell earlier than the mice of the control group did, with an average difference of 44 s. In the pole test, the mice in the MPTP group arrived on average earlier at the bottom than control mice did. The mice in the MPTP group did not grab the pole by their hind leg but slid down, which seemed to be caused by a lack of strength. This indicates that the MPTP mice showed decreased motor ability compared to that of control mice.

**FIGURE 2:**
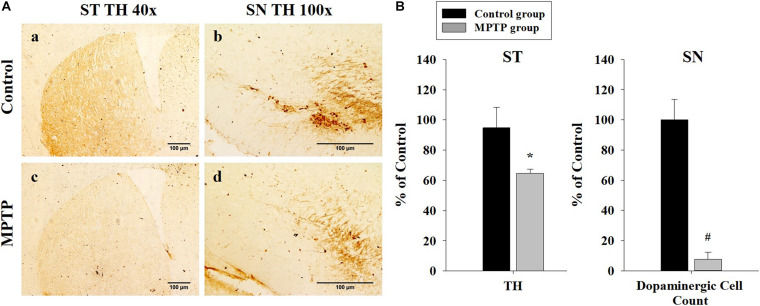
Immunohistochemistry analysis of tyrosine hydroxylase expression in the striatum and the substantia nigra (SN) in the control and 1-methyl-4-phenyl-1,2,3,6-tetrahydropyridine-treated (MPTP) groups. **(A)** Tyrosine hydroxylase staining is much stronger and darker in the striatum and the SN of the control (a, b) than that of the MPTP (c, d) mice because dopaminergic cells are destroyed in the SN of the MPTP mouse model. (scale bar, 100 μm). **(B)** The left graph shows the amount of tyrosine hydroxylase in the striatum, comparing panel a with panel c. The right graph shows the dopaminergic cell count in the SN, comparing the dopaminergic cell number in panel b with panel d. (*n* = 3, *P < 0.05, and ^#^*P* < 0.0005) Statistical analyses were carried out with Student’s *t*-test.

To ensure that the PD mouse model met the PD characteristics, TH expression was analyzed in the SN and the striatum using immunohistochemistry. The results showed that TH expression was reduced in both areas in the MPTP group. Furthermore, dopaminergic cell numbers decreased in the SN in the MPTP group (Figure 2). This decrease was caused by the destruction of dopaminergic cells by MPTP. This result indicates that the MPTP-induced PD model was well established.

**FIGURE 3:**
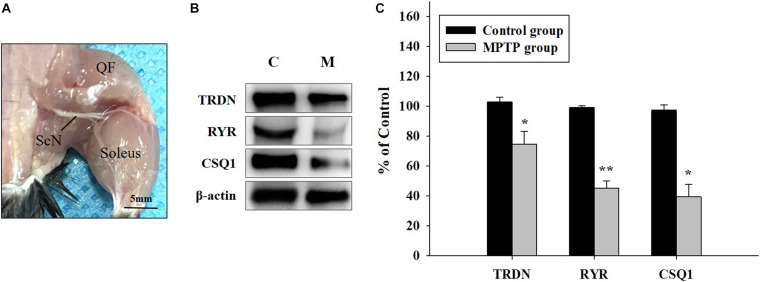
Western blot analysis of proteins linked to skeletal muscle contraction. **(A)** Tissue of the quadriceps femoris muscle, which is important for walking and jumping, was used in western blot analysis, as shown in **B**. (ScN, sciatic nerve). **(B)** Western blot analysis of triadin (TRDN), ryanodine receptor (RYR), and Calsequestrin 1 (CSQ1) which are factors of the Ca^2+^ channel in sarcoplasmic reticulum membrane from the control and 1-methyl-4-phenyl-1,2,3,6-tetrahydro pyridine treated (MPTP) groups. **(C)** Protein levels are shown as bar graphs from western blot results from **B**. (*n* = 3, *P < 0.05, and **P < 0.005) Statistical analyses were carried out with Student’s *t*-test.

Western blot analysis of QF muscle tissue (Figure 3A) showed that the expression levels of TRDN, RYR, and CSQ1 were reduced in the MPTP group (Figures 3B,C). RYR and CSQ1 levels were more than 50% lower compared to those in the control group. Considering that TRDN, RYR, and CSQ1 are components of a Ca^2+^ channel in the SR membrane of T-tubules, this could indicate reduced levels of Ca^2+^ channels in the SR membrane.

**FIGURE 4:**
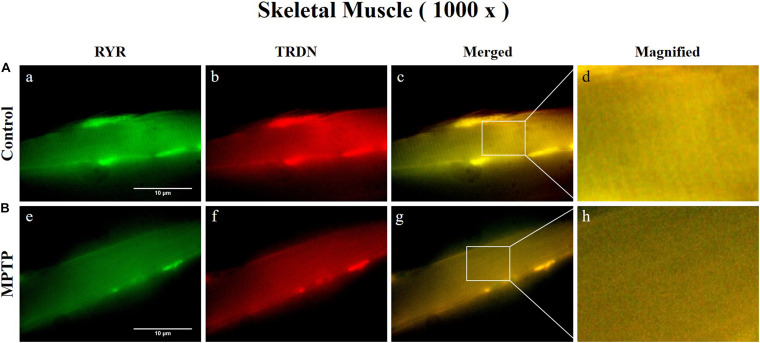
Immunofluorescence staining of ryanodine receptor (RYR) and triadin (TRDN) in skeletal muscle tissue (quadriceps femoris region). **(A)** Control group. a, RYR; b, TRDN; c, merge of a and b; and d, a 4 times magnified figure of a white rectangular box in c panel. **(B)** 1-methyl-4-phenyl-1,2,3,6-tetrahydropyridine treated (MPTP) group. e, RYR; f, TRDN; g, merge of e and f; and h, a 4 times magnified figure of a white rectangular box in g panel. (scale bar, 10 μm).

**FIGURE 5:**
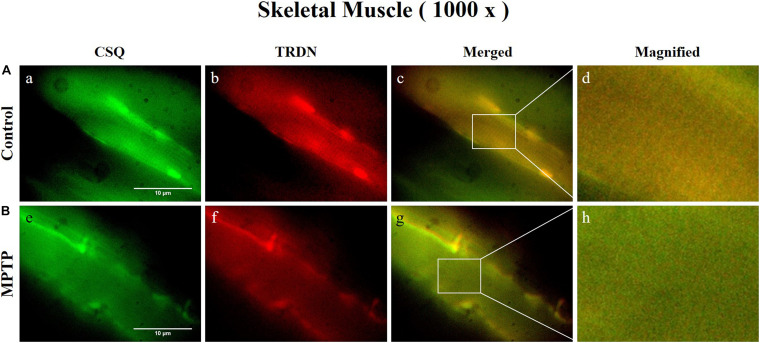
Immunofluorescence staining of Calsequestrin 1 (CSQ1) and triadin (TRDN) in skeletal muscle tissue (quadriceps femoris region). **(A)** Control group. a, CSQ1; b, TRDN; c, merge of a and b; and d, a 4 times magnified figure of a white rectangular box in c panel. **(B)** 1-methyl-4-phenyl-1,2,3,6-tetrahydropyridine treated (MPTP) group. e, CSQ1; f, TRDN; g, merge of e and f; and h, a 4 times magnified figure of a white rectangular box in g panel. (scale bar, 10 μm).

Immunofluorescence staining was carried out in longitudinal cryosections of the QF muscle to observe changes in RYR, CSQ1, and TRDN levels between the control group and the MPTP group. All processes were performed simultaneously under the same conditions. The results showed that the fluorescence intensity of RYR and TRDN decreased in the MPTP group (Figure 4), and the intensity of CSQ1 and TRDN also decreased in the MPTP group (Figure 5), which is consistent with the results shown in Figure 3. We hypothesized that the decrease in TRDN, RYR, and CSQ1 expression levels in skeletal muscle tissue would match the results at the cellular level.

**FIGURE 6:**
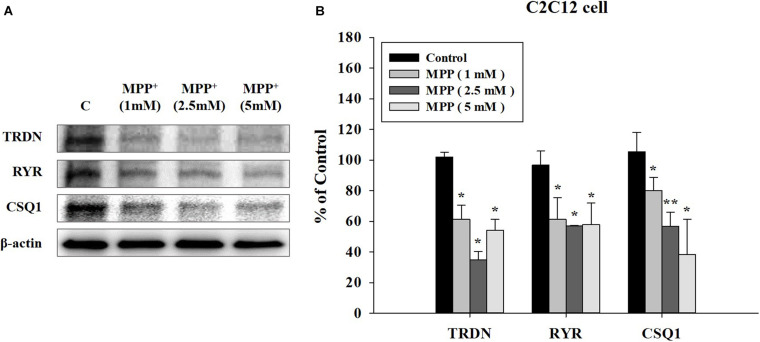
Western blot analysis of components linked to Ca^2+^ channels in sarcoplasmic reticulum at different 1-methyl-4-phenylpyridinium (MPP+) concentrations in C2C12 cells. **(A)** Western blot analysis of triadin (TRDN), ryanodine receptors (RYR), and Calsequestrin 1 (CSQ1), which are components of the Ca^2+^ channel in the sarcoplasmic reticulum membrane, at different MPP + treated concentrations: 1, 2.5, and 5 mM. MPP+ was applied for 18 h. **(B)** Quantified protein levels of the western blot results in **A**. (*n* = 3, *P < 0.05, and **P < 0.005) Statistical analyses were carried out with analysis of variance in SPSS 25.

To investigate the results at the cellular level, we used C2C12 mouse myoblast cells. After inducing the PD model with the neurotoxin MPP+, we examined the effect of MPP+ on C2C12 cells at increasing MPP + concentrations (Nicklas et al., 1987). Higher MPP + concentrations induced a larger decrease in the expression levels of the TRDN, RYR, and CSQ1 components of the Ca^2+^ channel in C2C12 cells (Figure 6).

**FIGURE 7:**
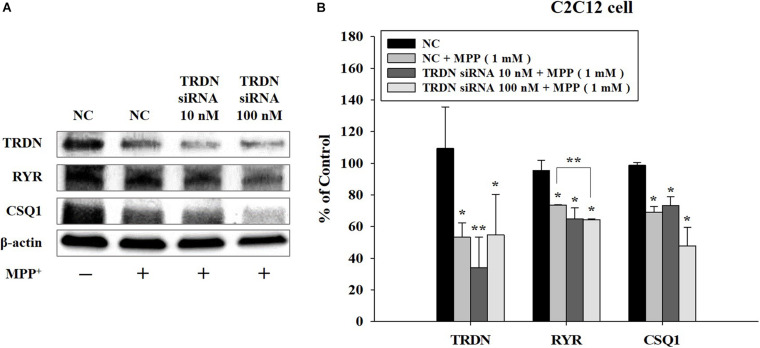
Western blot analysis of the components linked to Ca^2+^ channels in sarcoplasmic reticulum after treatment of C2C12 cells with 1-methyl-4-phenylpyridinium (MPP +) and triadin (TRDN) siRNA. **(A)** Western blot analysis of TRDN, ryanodine receptors, and Calsequestrin 1, which are proteins in the sarcoplasmic reticulum membrane, after MPP+ and TRDN siRNA application. Negative control cells were treated with negative control siRNA (100 nM) in the same condition as TRDN siRNA treatment. MPP+ was applied at a concentration of 1 mM for 18 h. The incubation time of TRDN and negative control siRNA was 48 h. **(B)** Quantified protein levels from the western blot results shown in **A**. Negative control (NC), negative control siRNA treatment (100 nM for 2 days); TRDN siRNA 10 nM, TRDN siRNA treatment (10 nM for 2 days); TRDN siRNA 100 nM, TRDN siRNA treatment (100 nM for 2 days). (*n* = 3, *P < 0.05, and **P < 0.005).

We also analyzed the changes in the components of the Ca^2+^ channel in C2C12 cells treated with MPP + when TRDN levels were decreased via TRDN siRNA (Figure 7). Because the protein levels of TRDN, RYR, and CSQ1 decreased in C2C12 cells treated with 1 mM MPP+ (Figure 6), MPP + treatment was performed equally with 1 mM in Figure 7. When 10 or 100 nM TRDN siRNA was added to MPP+ -treated C2C12 cells, the TRDN expression level also decreased. Furthermore, the expression levels of RYR and CSQ1 also decreased (Figure 7). This indicates that in this PD model, decreasing TRDN levels also decreased RYR and CSQ1 expression.

**FIGURE 8:**
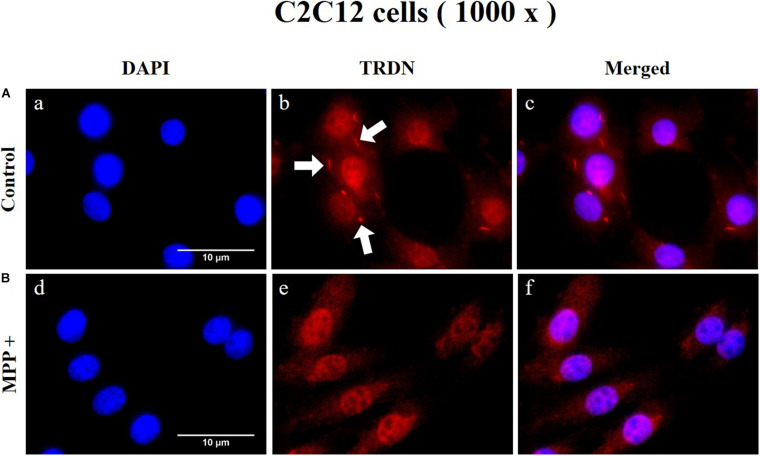
Immunofluorescence staining of triadin (TRDN) in C2C12 cells. **(A)** Control group. a, nuclei; b, TRDN; and c, merge of a and b. **(B)** 1-methyl-4-phenylpyridinium (MPP+) group. d, nuclei; e, TRDN; and f, merge of d and e. (scale bar, 10 μm).

To observe the change in TRDN expression at the cellular level according to MPP + treatment, immunofluorescence staining was performed in C2C12 cells in the control and MPP+ (1 mM)-treated group (Figure 8 and Supplementary Figure 1). As indicated by the arrows in Figures 8A, b, TRDN was clearly observable in the control group. However, TRDN was not observed in the MPP + treated group (Figures 8B, e). Thus, the decreased TRDN level in the PD model was verified once again.

## Discussion

In this study, we developed an MPTP-induced PD mouse model and used it to verify that the expression levels of Ca^2+^ channel components decreased in the leg QF muscle. In addition, we showed that the expression levels of TRDN, RYR, and CSQ1 were decreased by MPP + treatment in C2C12 cells at the cellular level. These results indicate that expression of the Ca^2+^ channel components TRDN, RYR, and CSQ1 were reduced in the PD model. Furthermore, the results showed that Ca^2+^ channel expression was decreased in the SR membrane of skeletal muscle. Because the Ca^2+^ channel related to RYR in the T-tubules is important for E-C coupling in muscle (Sutko and Airey, 1996), it is considered that the results of this study showing decreased RYR Ca^2+^ channel expression would be important in PD. As Ca^2+^ release from the SR lumen in the muscle is important for contraction, insufficient Ca^2+^ release because of decreased Ca^2+^ channel expression could impair the adjustment of muscle contraction. This might be linked to bradykinesia (slow movement), one of the symptoms seen in patients with PD. However, it is necessary to further investigate the factors related to Ca^2+^ homeostasis regulation.

In addition, CSQ1 is known as a luminal regulator of RYR activity and inhibits the Ca^2+^ release channels (Beard et al., 2004). The lower expression of CSQ1 in our PD model (Figures 3, 5) may induce Ca^2+^ leakage from the SR that could delay the restoration of the Ca^2+^ concentration in the SR lumen during contraction. TRDN expression was also decreased in the PD model (Figures 3, 6), resulting in poor Ca^2+^ regulation in the SR because one of the functions of TRDN is the regulation of Ca^2+^ release from the SR, and the loss of TRDN leads to RYR Ca^2+^ leakage. These changes demonstrate that the function of the Ca^2+^ release channel is impaired or that the number of Ca^2+^ release channels decreases, which impairs Ca^2+^ release from the SR lumen and leads to symptoms related to muscle function in PD, such as bradykinesia or tremor.

To test whether TRDN is able to regulate CSQ1 expression (Oddoux et al., 2009), we used TRDN siRNA knockdown in our PD model (Figure 7). This reduction in TRDN induced a decrease in RYR and a tendency to decrease CSQ1 (Figure 7). Therefore, TRDN might be an important factor in the regulation of Ca^2+^ release from the SR in PD. It is anticipated that restoring decreased TRDN levels could alleviate the symptoms of PD, such as bradykinesia, tremor, or walking impairments. This is meaningful for improving the quality of life of patients with PD. However, other Ca^2+^ homeostasis regulation factors and the precise regulation mechanisms will need to be investigated in future studies. Furthermore, it is reported that dysregulated Ca^2+^ homeostasis in dopaminergic neuron leads to impaired mitochondria (Cali et al., 2014) and alterations of Ca^2+^ homeostasis including depletion of endoplasmic reticulum Ca2 + storage are implicated with neurodegenerative process (Mattson, 2012; Schapira, 2013; Zaichick et al., 2017). In this regard, the alteration of triadic proteins in neurons related to Ca^2+^ channel in endoplasmic reticulum of dopaminergic neuron would be prospective research direction and the changes of mitochondria in muscle related to Ca^2+^ homeostasis would be also interesting research in a forward study.

In conclusion, the expression levels of RYR, TRDN, and CSQ1, which are components of the Ca^2+^ channel related to E-C coupling, decreased in the QF muscle of MPTP-induced PD mice and in C2C12 cells treated with MPP+. Decreasing TRDN levels reduced RYR and CSQ1 levels and this might cause a Ca^2+^ leak via RYRs and a decrease in Ca^2+^ release channel levels. In addition, this would delay Ca^2+^ uptake in the SR lumen and muscle contraction until the necessary Ca^2+^ concentration is reached in the cytoplasm, potentially linking this mechanism to PD symptoms. These results suggest that the levels of proteins related to Ca^2+^ channel function decreased in this model, which could impair muscle function. We conclude that muscle function alterations could add to the bradykinesia and tremor in this model of PD. Restoring the components of the Ca^2+^ channel investigated in this study might relieve the symptoms related to muscle function in PD.

## Data Availability Statement

The data will be available upon request to the corresponding author.

## Ethics Statement

The animal study was reviewed and approved by Institutional Animal Care and Use Committee (IACUC) of Sangji University.

## Author Contributions

SY and MS: conceptualization. SY and MS: methodology. MS: software. SY and MS: validation. SY and MS: formal analysis. MS: investigation. SY: resources. MS: data curation. MS: writing—original draft preparation. SY: writing—review and editing. MS: visualization. SY: supervision. SY: funding acquisition. Both authors have read and agreed to the published version of the manuscript.

## Conflict of Interest

The authors declare that the research was conducted in the absence of any commercial or financial relationships that could be construed as a potential conflict of interest.
