# Supplementary material for: Triadin Decrease Impairs the Expression of E-C Coupling Related Proteins in Muscles of MPTP-Induced Parkinson’s Disease Mice
Source: Front Neurosci. 2021 Apr 22;15:649688. doi: 10.3389/fnins.2021.649688 (PMC8100520; doi:10.3389/fnins.2021.649688)
Supplement: Supplementary file 1 [file Data_Sheet_1.PDF]

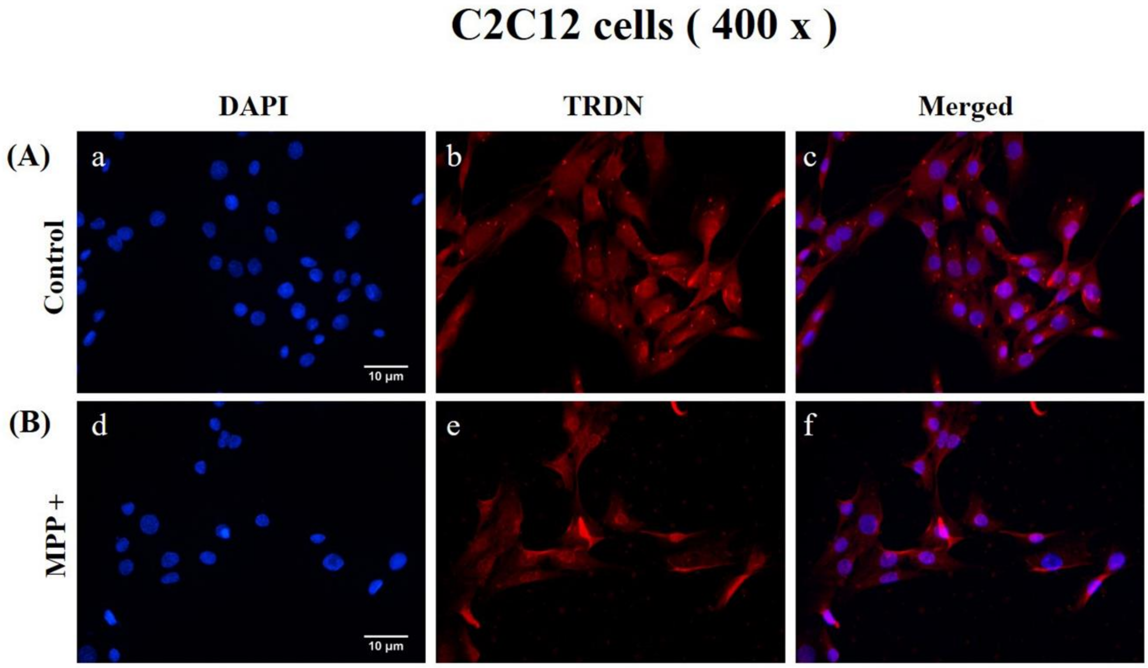


**Supplementary figure 1. Immunofluorescence staining of triadin (TRDN) in C2C12 cells. (A)** Control group. a, nuclei; b, TRDN; c; merge of a and b. **(B)** 1-methyl-4-phenylpyridinium **(**MPP+) group. d, nuclei; e, TRDN; f, merge of d and e. (scale bar, 10 μm)
